# Selective degradation of IKKα by autophagy is essential for arsenite-induced cancer cell apoptosis

**DOI:** 10.1038/s41419-020-2420-5

**Published:** 2020-04-07

**Authors:** Qixing Tan, Shuxian Zou, Rui Jin, Yongliang Hu, Huan Xu, Hongli Wang, Mengnan Ding, Meiru Hu, Changyuan Wei, Lun Song

**Affiliations:** 1 Institute of Military Cognitive and Brain Sciences, 27 Taiping Road, Beijing, 100850 P. R. China; 2 Department of Breast Surgery, Guangxi Medical University Tumor Hospital, 71 Hedi Road, Nanning, 530021 P. R. China; 3 Department of Tumor Biology, Beijing Institute of Biotechnology, 27 Taiping Road, Beijing, 100850 P. R. China; 4 Anhui Medical University, 81 Meishan Road, Hefei, 230032 P. R. China; 5 Present Address: Department of Dermatology, The 8th Medical Center of Chinese PLA, 17 Heishanhu Street, Beijing, 100091 P. R. China

**Keywords:** Macroautophagy, Apoptosis

## Abstract

Two catalytic subunits of the IKK complex, IKKα and IKKβ, trigger NF-κB activation as well as NF-κB-independent signaling events under both physiological and pathological conditions. Here we identified the NF-κB-unrelated cytoprotective function of IKKα in promoting autophagy by triggering p53 transactivation and upregulation of its downstream autophagic mediator, DRAM1, in the arsenite-treated hepatoma cells, which responses depended on IKKα kinase activity. Furthermore, IKKα triggered p53/DRAM1-dependent autophagy by inducing CHK1 activation and CHK1/p53 interaction. Interestingly, after provoking autophagy, IKKα could be specifically recognized by the autophagic machinery via directly binding with LC3B, resulting in selective degradation of IKKα by autophagy. Unexpectedly, the selectivity of autophagic sequestration towards IKKα was mediated by novel mechanism independent of the classical LC3-interacting regions (LIRs) within IKKα, while C-terminal arm of LIR was involved in mediating IKKα/LC3B interaction. Taken together, we conclude that IKKα attenuates arsenite-induced apoptosis by inducing p53-dependent autophagy, and then selective feedback degradation of IKKα by autophagy contributes to the cytotoxic response induced by arsenite.

## Introduction

Autophagy is an evolutionarily conserved and highly complex catabolic process that mainly targets cytoplasmic components (macromolecules and organelles) for degradation via the lysosomal pathway. In mammals, three modes of autophagy have been identified: macroautophagy, microautophagy, and chaperone-mediated autophagy (CMA). These three modes differ with respect to the pathway by which cytoplasmic material is delivered to the lysosome, but share in common the final steps of lysosomal degradation of the cargo. Usually, macroautophagy is initiated by the formation of a vacuole called an autophagosome that ultimately fuses with lysosomes where the sequestered material is degraded; while microautophagy and CMA do not require de novo synthesis of autophagosomes to transport cargo to the lysosome or vacuole. Autophagy not only plays a critical role in maintaining intercellular homeostasis, but also is considered a mediator of cellular processes under various stress conditions and highly relevant to a number of diseases^1–3^.

Macroautophagy (hereafter referred to autophagy) was traditionally regarded as a non-selective, bulk degradation process mainly induced to keep up with the energy demand upon starvation. However, growing evidence support that autophagic degradation is also highly selective. The selectivity of autophagic sequestration towards intracellular pathogens, damaged organelles, cellular structures, protein aggregates or specific soluble proteins are important for cell functions^4,5^. The discovery and characterization of autophagic cargo receptors such as SQSTM1/p62, NBR1, NDP52, Optineurin, NIX, etc., has provided mechanistic insight into selective autophagy. These cargo receptors contain both LC3- interacting region (LIR) and the cargo interacting (ubiquitin-dependent or ubiquitin-independent) domain, which enable them to specifically recognize the substrates and simultaneously bind to the lipidated LC3 or other ATG8 family members and therefore facilitate docking of the selective substrates to the autophagosome^6,7^. In addition, some reports have characterized another mechanism for the selective degradation of cellular proteins via the autophagic clearance pathway, by which proteins can directly interact with LC3 or other ATG8 family members through their existing classical LIR motif (W/F/Y-X-X-V/L/I) without the aid of autophagic cargo receptors^8,9^.

The IκB kinase (IKK) complex, consisting of two catalytic subunits (IKKα and IKKβ) and one regulatory subunit (IKKγ), play a critical role in the activation of the NF-κB pathways under both physiological and pathological conditions. IKKα and IKKβ share structural similarity but trigger NF-κB activation by different mechanisms^10^. Furthermore, both IKKα and IKKβ are demonstrated to possess some unique functions that are independent to NF-κB activity, but are mediated by NF-κB-unrelated substrates, such as Aurora A, Maspin, 14-3-3σ, FOXO3, SMRT, p53, SRC3, c-Fos, p85α, mTOR, MDM2, and ATG16L1^10–12^. Therefore, it is believed that both IKKα and IKKβ can act as multifunctional signaling proteins with roles going far beyond their well-known action in NF-κB pathway regulation.

In our previous reports, we demonstrate that both IKKα and IKKβ have the ability to mediate stress responses through NF-κB-independent mechanisms. Moreover, some specificity occurs between IKKα and IKKβ, because their substrates are exclusively regulated by one kinase but not the other^13–17^. In the current study, we revealed that IKKα played a key role in inducing autophagy in response to the treatment of cytotoxic chemical reagents, arsenite. Notably, this effect of IKKα was unrelated to NF-κB activity, but was delivered by the activation of CHK1/p53/DRAM1 pathway. Furthermore, IKKα could be selectively degraded by autophagy via directly interacting with LC3B after activation, and this feed-back control of IKKα is critical for mediating the pro-apoptotic effect of arsenite.

## Materials and methods

### Plasmids, antibodies, and reagents

The NF-κB and p53-dependent luciferase reporter plasmids, the constructs expression FLAG-IKKα and FLAG-IKKα-KM were described in our previous reports^14,17^. The following primary antibodies were purchased from Cell Signaling Technology (Beverly, MA, USA): Beclin-1, LC3B, SQSTM1, ATG5, phospho-p53-Ser15, p53, phospho-IKKα-Ser176/180, IKKα, IKKβ, IκBα, phospho-CHK1-Ser345, CHK1, FLAG, and ACTB. The following primary antibodies were purchased from Santa Cruz Biotechnology (Santa Cruz, CA, USA): IKKγ, GADD45α, and DRAM1. *IKKα* siRNA, *p53* siRNA, and *ATG5* siRNA were purchased from Cell Signaling Technology (Beverly, MA, USA). *DRAM1* siRNA and *CHK1* siRNA were purchased from Riobo Technology (Guangzhou, China). 3-MA, BafA1, and MG132 were ordered from Sigma-Aldrich (St. Louis, MO, USA).

### Generation of human IKKα mutant constructs

The following deletion and point mutants of IKKα were constructed by using in vitro site-directed mutagenesis system (Nuoweizan Biotechnology, China): IKKα deleting 213-FECI-216 (IKKαΔLIR1), IKKα deleting 276-WLQL-279 (IKKαΔLIR2), IKKα with point mutation on the N- or C-terminal arms of putative LIR1 and LIR2 (IKKα-V211A, IKKα-A216T, IKKα-Y218F, IKKα-P271R, IKKα-N274K, IKKα-N281M, IKKα-D283H), The amino acids in IKKα point mutation were mutated to the corresponding ones in IKKβ.

### Cell culture and transfection

HepG2 human hepatoma cells were maintained in DMEM with 10% fetal bovine serum supplemented with antibiotic/antimycotic. Analysis was performed to exclude the mycoplasma contamination. Transfections were performed with the LipofectAMINE 2000 or LipofectAMINE^TM^ RNAi MAX (Invitrogen) according to the manufacturer’s instructions.

### Immunoprecipitation and immunoblot assay

HepG2 cells were left untreated or treated with arsenite and then reciprocal immunoprecipitations (IPs) were performed to detect the endogenous IKKα/LC3B, IKKα/p53, IKKα/CHK1 or CHK1/p53 interaction. The detected whether IKKα is required for CHK1/p53 interaction in the arsenite response, HepG2 cells were transfected with *IKKα* siRNA or the control siRNA, and then reciprocal IPs were performed to detect the changes on CHK1/p53 interaction with or without IKKα expression. Cellular protein preparation and immunoblot assays were performed as described previously^17,18^.

### Luciferase reporter assay

Cells were cotransfected with an experimental reporter (either a p53- or NF-κB-dependent luciferase reporter), a control reporter (Renilla luciferase reporter), and then the stable transfectants were established. Luciferase activity was tested at 12 h after arsenite exposure using Firefly-Renilla Dual-Luciferase Reporter Assay System (Promega). The data were obtained by normalizing the activity of the experimental reporter to that of the internal control. The results were presented as the relative induction by normalizing the luciferase activity in the arsenite-treated cells to the luciferase activity in untreated control cells, as previously described^17,18^.

### RNA isolation and RT-PCR assay

Total RNA was extracted with TRIzol reagent (Sigma-Aldrich), and cDNA was synthesized with the ThermoScriptTM RT-PCR system (Thermo Fisher Scientific). To analyze the transcription of *IKKα*, *IKKβ* and *DRAM1*, the specific primers (can be obtained if required) were designed to amplify the target cDNAs.

### Immunofluorescence assay

To detect the subcellular distribution of IKKα and LC3B, HepG2 cells with or without arsenite exposure were fixed and then incubated with the primary antibodies against the different targets and the FITC or PE-conjugated secondary antibodies. The signal was monitored using the confocal microscopy (ZEISS, LSM510 META).

### Cell apoptosis assay

Arsenite-induced apoptosis in HepG2 cells was determined by propidium iodide (PI) staining of nuclei as described in our previous reports^17^.

### Autophagy assay

Cellular autophagy was monitored using the following techniques: western blot analysis of specific key proteins (increase in the endogenous LC3BII: I ratio, upregulation of Beclin-1 expression and dynamic degradation of SQSTM1), confocal microscopy (ZEISS, LSM510 META) and flow cytometry (BD Biosciences, FACSCalibur)-based quantitative analysis. The Cyto-ID Autophagy Detection Kit (Enzo Life Sciences) was used to monitor specific autophagic fluorescence signals under confocal microscopy or to quantitatively measure the autophagic fluorescence intensity by flow cytometric analysis as previously described^18^. In addition, BafA1 was used to assess the induction of autophagic flux also as described previously^18^.

### Statistics

To determine the effect of a single treatment within a group, Student’s *t*-test was used to test the significance of the data. To determine the effects of treatment × group interactions, factorial design (AVONA) was employed to test the significance of the data. At least three independent experiments were performed. For the judgment of data validity, we took the standard deviation (SD) less than one third of the average as the standard. The group allocation was totally blinded to the detector. The results were presented as the mean ± SD. The level of significance was set at *P* < 0.05.

## Results

### Downregulation of IKKα was required for mediating apoptosis induced by arsenite, which response was unrelated to NF-κB transactivation

In the previous reports, we demonstrate that GADD45α accumulation is the critical signaling event in mediating arsenite-induced cancer cell apoptosis, which response is accompanied by downregulation of both the catalytic subunits of the IKK complex, IKKα, and IKKβ. Then we reveal that IKKβ reduction results from the transcriptional repression and exerts a novel NF-κB-independent function in modulating GADD45α protein stability and cell apoptosis^17^. However, the signaling events leading to the suppression of IKKα expression and its subsequent biological significance in the arsenite responses have not been clarified.

**Fig. 1:**
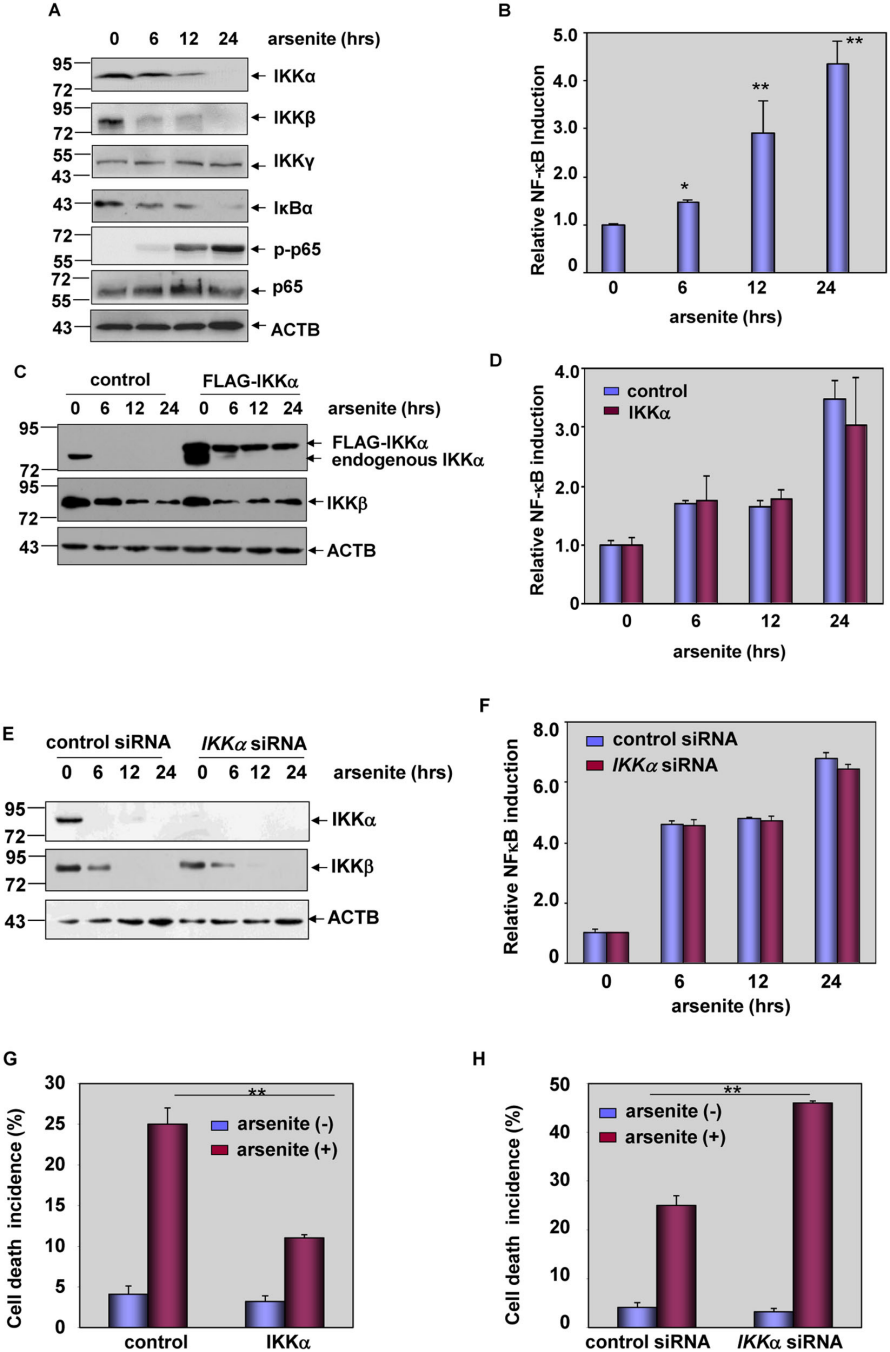
Downregulation of IKKα was required for mediating apoptotic effect but was unrelated to NF-κB transactivation under arsenite exposure. **a** HepG2 cells were treated with arsenite (20 μM) for the indicated time periods and then the levels of IKKα, IKKβ, IKKγ, IκBα and the activation of p65 were detected. **b** HepG2 cells were transfected with NF-κB-dependent luciferase reporter and the stable transfectants were established. The transfectants were exposed to arsenite (20 μM) for the indicated time period and then the induction of NF-κB-dependent luciferase activity was examined (**P < 0.01, *P < 0.05). **c** HepG2 cells were transfected with expression plasmids encoding FLAG-IKKα or the control vector and then subjected to arsenite (20 μM) exposure. The expression levels of IKKα and IKKβ were detected at 12 h after arsenite treatment. **d** HepG2 cells stably transfected with NF-κB-dependent luciferase reporter were transfected with FLAG-IKKα construct or the control vector and then exposed to arsenite (20 μM). The induction of NF-κB-dependent luciferase activity was determined at the indicated time periods after arsenite exposure. **e** HepG2 cells were transfected with siRNA specifically targeting *IKKα* or the control siRNA and then treated as described in (**c**). The detections were also performed as described in (**c**). **f** HepG2 cells stably transfected with NF-κB-dependent luciferase reporter were transfected with *IKKα* siRNA or the control siRNA and then treated as described in (**d**). The detections were also performed as described in (**d**). **g**, **h** HepG2 cells were transfected and treated as described in (**c**) and (**e**). The cell death incidence was detected by flow cytometric assay at 24 h after arsenite exposure (**P < 0.01).

Here we repeatedly found that downregulation of IKKα and IKKβ was accompanied by NF-κB transactivation, evidenced by a time-dependent degradation of IκBα, phosphorylation of p65, and the upregulation of NF-κB-dependent luciferase activities in the arsenite-treated HepG2 hepatoma cells (Fig. 1a, b). Most importantly, the induction of NF-κB-dependent luciferase activities did not show detectable changes before and after IKKα overexpression or depletion (Fig. 1c–f), indicating that IKKα reduction is also a signaling event unrelated to NF-κB transactivation. Then, we observed that overexpression of IKKα in HepG2 cells attenuated cell death incidence, while knockdown of IKKα expression increased the percentage of dead cells in response to arsenite stimulation (Fig. 1g, h). These results indicate that IKKα functions as a protector in arsenite-induced pro-apoptotic responses. Thus, reduction of IKKα is essential for mediating apoptosis in HepG2 cells.

### IKKα underwent autophagy-dependent degradation upon arsenite exposure

**Fig. 2:**
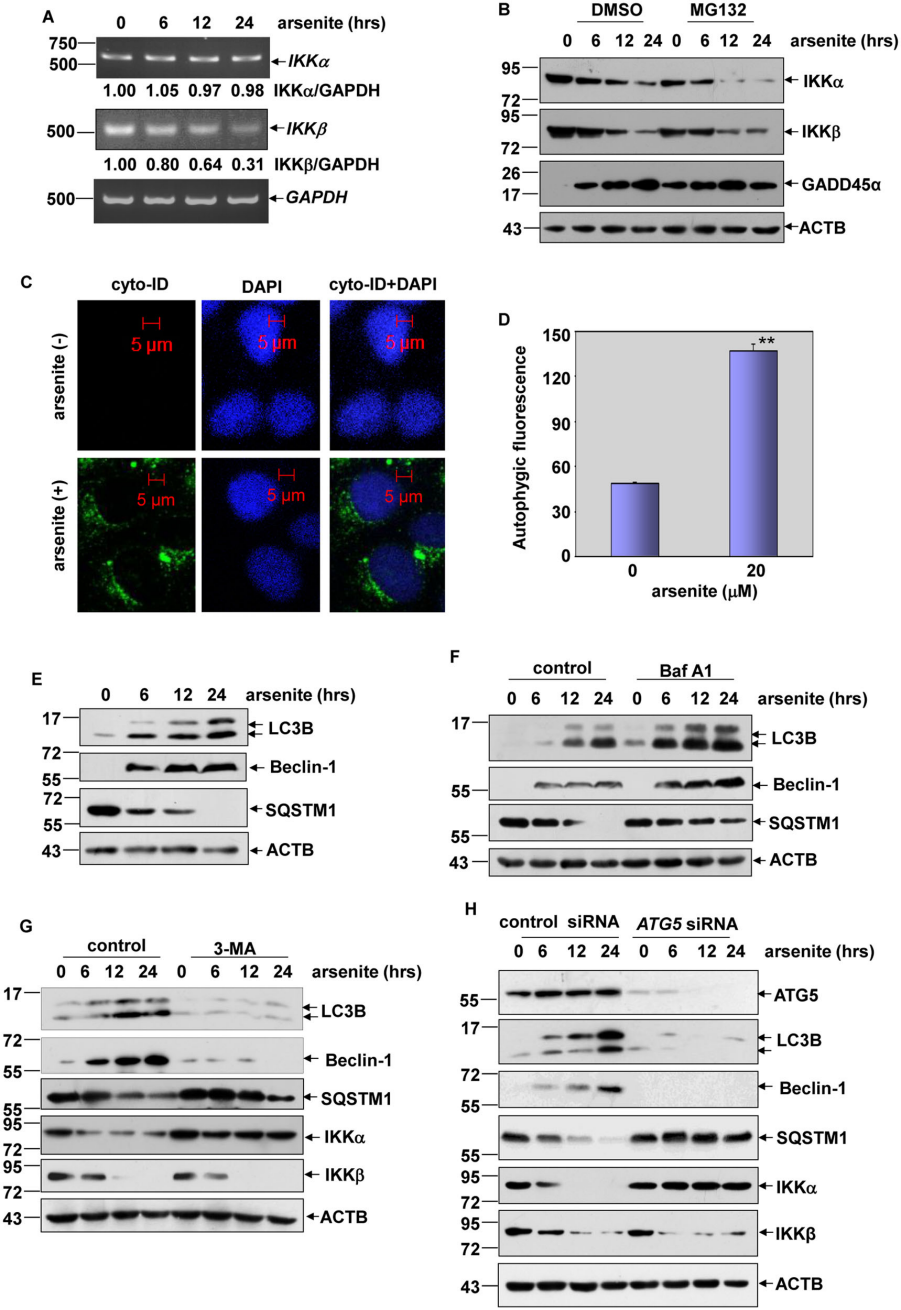
Autophagy-dependent degradation of IKKα resulted to its downregulation under arsenite exposure. **a** HepG2 cells were treated with arsenite (20 μM) for the indicated time periods and then *IKKα* and *IKKβ* mRNA levels were detected. **b** HepG2 cells were pretreated with MG132 (5 μM) followed by exposure to arsenite (20 μM). Then the levels of GADD45α, IKKα, and IKKβ were detected. **c** HepG2 cells were left untreated or were treated with arsenite (20 μM) for 24 h; then, autophagy was examined under confocal microscopy after the cells were stained with Cyto-ID Green Autophagy Detection Reagent. **d** HepG2 cells were treated with arsenite and were stained with Cyto-ID Autophagy Detection Reagent as described in (**c**). Then, the cells were collected and subjected to a flow cytometric analysis to quantitatively measure the autophagic fluorescence intensity inside the cells (**P < 0.01). **e** HepG2 cells were treated with arsenite (20 μM) for the indicted time periods, and then expression levels of LC3B, Beclin-1 and SQSTM1 were examined at the indicated time periods after arsenite exposure. **f** HepG2 cells were treated with arsenite (20 μM) alone or in combination with BafA1 (0.1 μM) during the final 4 h before the cells were harvested. Then, the expression levels of LC3B, Beclin-1, and SQSTM1 were examined at 12 h after arsenite exposure. **g** HepG2 cells were pretreated with 3-MA, followed by exposure to arsenite (20 μM). Then, the expression of IKKα, IKKβ, LC3B, Beclin-1, and SQSTM1 was analyzed at 12 h after arsenite exposure. **h** HepG2 cells were transfected with *ATG5* siRNA or control siRNA and then exposed to arsenite (20 μM) 36 h after transfection. The expression of ATG5, IKKα, IKKβ, LC3B, Beclin-1, and SQSTM1 was examined at 12 h after arsenite exposure.

Next, we focused on investigating the signaling events leading to IKKα reduction under arsenite exposure. Here we found that downregulation of IKKβ expression in HepG2 cells was accompanied by transcriptional suppression of *IKKβ*, while IKKα reduction does not result from the inhibition of *IKKα* mRNA transcription (Fig. 2a). Then we asked whether IKKα reduction involved ubiquitin and proteasome-dependent degradation. However, arsenite-induced dynamic changes on IKKα expression were similar with or without the pretreatment of MG132, the proteasome inhibitor (Fig. 2b). The effectiveness of MG132 on blocking proteasome-dependent degradation pathway was confirmed by the accumulation of GADD45α, which constitutively degraded via proteasome-dependent manner^13,17^, after MG132 treatment (lanes 1 and 5 in GADD45α panel in Fig. 2b). Furthermore, we didn’t observe the signal for ubiquitination of IKKα in the absence or presence of arsenite exposure (data not shown). These data together thus exclude the possibility of proteasome-dependent degradation to IKKα after arsenite exposure.

Then, we addressed whether IKKα reduction results from autophagy-dependent degradation. As shown in Fig. 2c, when HepG2 cells were stained with Cyto-ID Autophagy Detection Reagent (provided in the Cyto-ID Autophagy Detection Kit), we observed a significant induction of autophagic activity inside the cells after arsenite exposure, which was evidenced by a specific green autophagic fluorescence signal that accumulated in spherical vacuoles in the perinuclear region of arsenite-treated cells. Furthermore, according to the results form the flow cytometry-based quantitative analysis of cell populations loaded with the Cyto-ID, the autophagic fluorescence signals increased dramatically in HepG2 cells after exposure to arsenite for 24 h (Fig. 2d). Collectively, these data indicate that arsenite exposure induces autophagic activity in HepG2 cells. Next, we analyzed the levels of LC3B, Beclin-1 and SQSTM1, the hallmarks of autophagosome accumulation and autophagic degradation, in the arsenite-treated HepG2 cells. We found that arsenite exposure resulted in a time-dependent increase in the LC3B-II/I ratio, an induction of Beclin-1 expression and a decrease in SQSTM1 levels (Fig. 2e), indicating the enhanced autophagosome synthesis and the activation of autophagic degradation pathways in response to arsenite stimulation. In addition, we also found a significantly higher accumulation of LC3B and Beclin-1 and a rescue of SQSTM1 degradation under the co-treatment of arsenite and BafA1compared with arsenite treatment alone (Fig. 2f). These data clearly demonstrate that arsenite exposure results in an increase in autophagic flux, rather than defects in autophagic degradation, in HepG2 cells.

Finally, we examined whether IKKα reduction involved autophagic degradation. As shown in Fig. 2g, HepG2 cells were left untreated or pretreated with 3-MA, followed by exposure to arsenite. The efficiency of 3-MA in inhibiting arsenite-induced autophagy was verified by the reduction in LC3B and Beclin-1 expression and rescue of SQSTM1 degradation in the 3-MA and arsenite co-treated HepG2 cells compared to the cells treated with arsenite alone. Then, we found that reduction in IKKα expression was almost completely blocked by 3-MA pretreatment, while transcriptional suppression of IKKβ did not show detectable changes with or without 3-MA pretreatment. Furthermore, knockdown of ATG5 expression not only interrupted the induction of LC3B and Beclin-1 expression and SQSTM1 degradation but also blocked IKKα reduction induced by arsenite; while suppression of IKKβ expression did not change obviously with or without ATG5 expression (Fig. 2h). These results indicate that selective degradation of IKKα by autophagic pathway results in its downregulation under arsenite exposure.

### Direct binding with LC3B was critical for selective degradation of IKKα by autophagic pathway in response to arsenite exposure

**Fig. 3:**
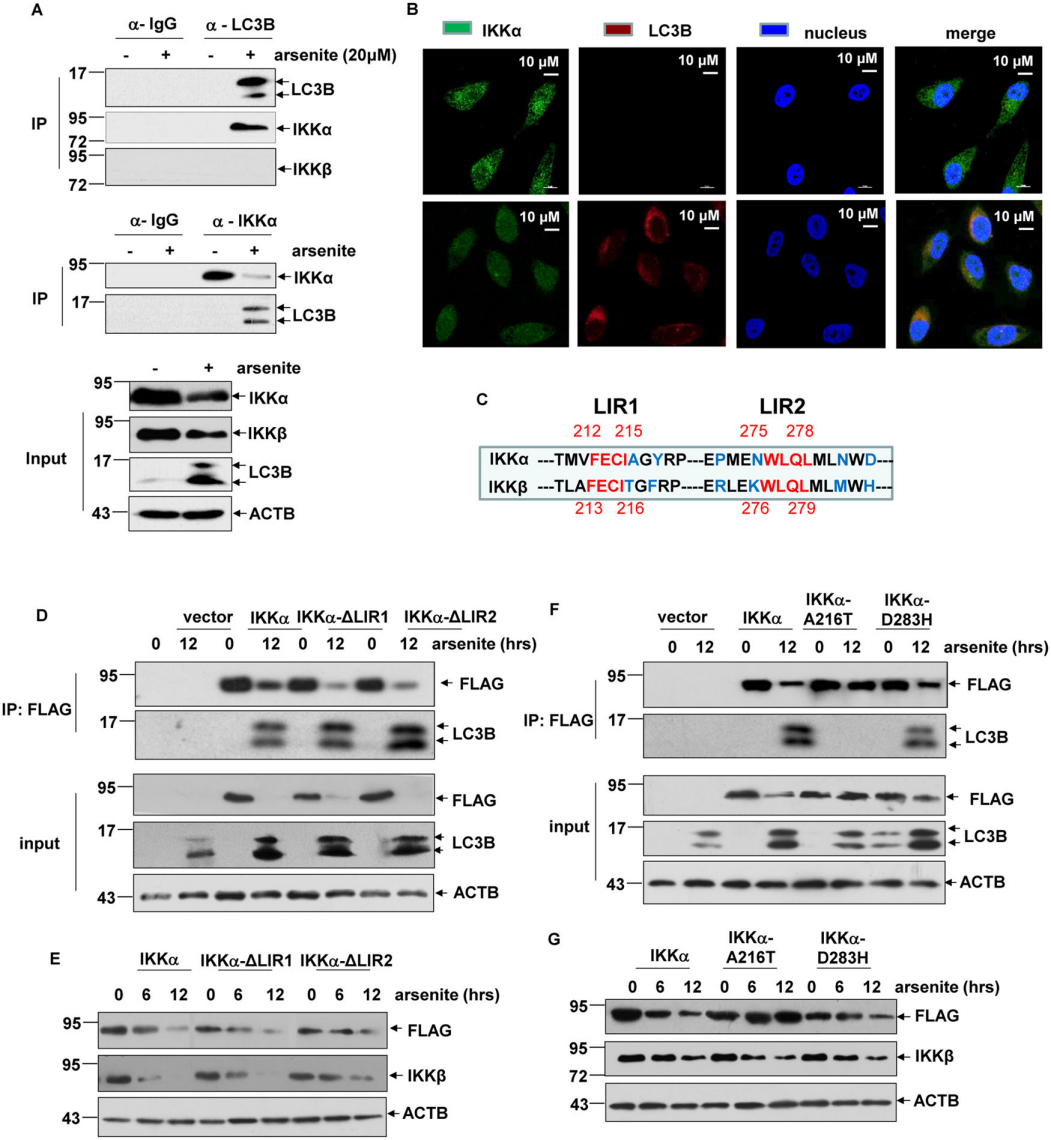
Interaction with LC3B was critical for autophagic degradation of IKKα in response to arsenite exposure. **a** HepG2 cells were left untreated or treated with arsenite (20 μM) for 8 h and then the interaction between endogenous IKKα, IKKβ and LC3B was detected by immunoprecipitation assay. **b** HepG2 cells were left untreated or treated with arsenite (20 μM) for 8 h. Then the subcellular distributions of IKKα and LC3B were detected by immunofluorescence assay. **c** The two clusters of sequences in IKKα and IKKβ matched to the classical LIR were identified. The sequences of N- and C-terminal arms of LIR in IKKα showed large differences with the corresponding sequences in IKKβ were also indicated. **d** HepG2 cells were transfected with the constructs expressing wild-type IKKα, IKKα∆LIR1 or IKKα∆LIR2 followed by exposure to arsentie, and then their binding abilities to LC3B were detected by co-IP. **e** HepG2 cells were transfected as describe in (**d**) and then exposed to arsentie for the indicated time periods. The degradation dynamics of wild-type IKKα and its mutants were detected. **f** HepG2 cells were transfected with the constructs expressing wild-type IKKα, IKKα-A216T or IKKα-D283H followed by exposure to arsentie, and then their binding abilities to LC3B were detected by co-IP. **g** HepG2 cells were transfected as described in (**f**) and then exposed to arsentie for the indicated time periods. The degradation dynamics of wild-type IKKα and its mutants were detected.

Next, we tried to figure out why IKKα but not IKKβ was able to be subjected to the autophagy-dependent degradation pathway. Since interaction with LC3B is one of the mechanisms contributing to the selective degradation of target proteins by autophagic pathway^8,9^, we thus firstly detected the potential binding ability of IKKα and IKKβ with LC3B. As shown in Fig. 3a, arsenite exposure induced a strong interaction between endogenous IKKα and LC3B according to the results from the reciprocal immunoprecipitaiton assay. However, no signal indicating IKKβ/LC3B interaction was observed under the same conditions. In the following immunofluorescence assays, we further observed co-localization of IKKα and LC3B within the cytoplasm after a short period of arsenite exposure (before IKKα signal dropped sharply), evidenced by the significant overlapping signals for cytoplasmic IKKα/LC3B (Fig. 3b). These results further confirmed the recruitment of IKKα in the autophagosome.

Then, we tried to figure out the structural basis involved in IKKα/LC3B complex formation. Based on the data from the sequence analysis of human IKKα protein, two clusters of sequences perfectly matched to classical LIR motif (W/F/Y-X-X-V/L/I) were identified (212-FECI-215 and 275-WLQL-278) (Fig. 3c). Therefore, IKKα mutants deleting the putative LIR sequences (IKKαΔLIR1 and IKKαΔLIR2) were constructed. However, we found that deleting the putative LIR1 and LIR2 in IKKα did not affect its binding ability to LC3B (Fig. 3d). In addition, degradation dynamics of the IKKαΔLIR1 and IKKαΔLIR2 mutants did not change obviously compared with the wild-type counterpart (Fig. 3e). In fact, human IKKβ consists of the exactly same potential LIR motifs (213-FECI-216 and 276-WLQL-279) (Fig. 3c), but IKKβ failed to interact with LC3B and did not undergo autophagic degradation upon arsenite exposure (Figs. 3a, 2g, h). Taken together these results, we excluded the contribution of the potential LIR motifs within IKKα to binding to LC3B and autophagic degradation of IKKα in the arsenite responses.

Then we asked whether the N- and C-terminal arms of LIRs in IKKα, which showed great differences with the corresponding sequences in IKKβ (the blue amino acids in Fig. 3c), play any role in determining IKKα/LC3B complex formation. To this end, a series of IKKα point mutants, in which the amino acids in the N- and C-terminal arms of LIRs were mutated to the corresponding ones in IKKβ, were constructed. As shown in Fig. 3f, mutagenesis of Alanine 216 in IKKα to Thronine (the corresponding amino acid in IKKβ) completely deprived IKKα of its binding ability to LC3B, while other IKKα point mutants (IKKα-Y218F, IKKα-P271R, IKKα-N274K, IKKα-N281M, IKKα-D283H) exhibited the similar LC3B binding ability as their wild-type counterpart (only the data from IKKα-D283H was shown here as a representative result). Additionally, IKKα-A216T kept stable after long term of arsenite exposure, while IKKα-D283H and other IKKα point mutants exhibit the similar degradation dynamics as wild-type IKKα under the same arsenite exposure conditions (Fig. 3g and data not shown). Taken together, these data indicate that Ala 216 in IKKα is essential for mediating IKKα/LC3B interaction and therefore played a critical role in determining the selective degradation of this protein by autophagy-dependent pathway.

### p53 transactivation was responsible for autophagy-dependent degradation of IKKα under arsenite exposure

**Fig. 4:**
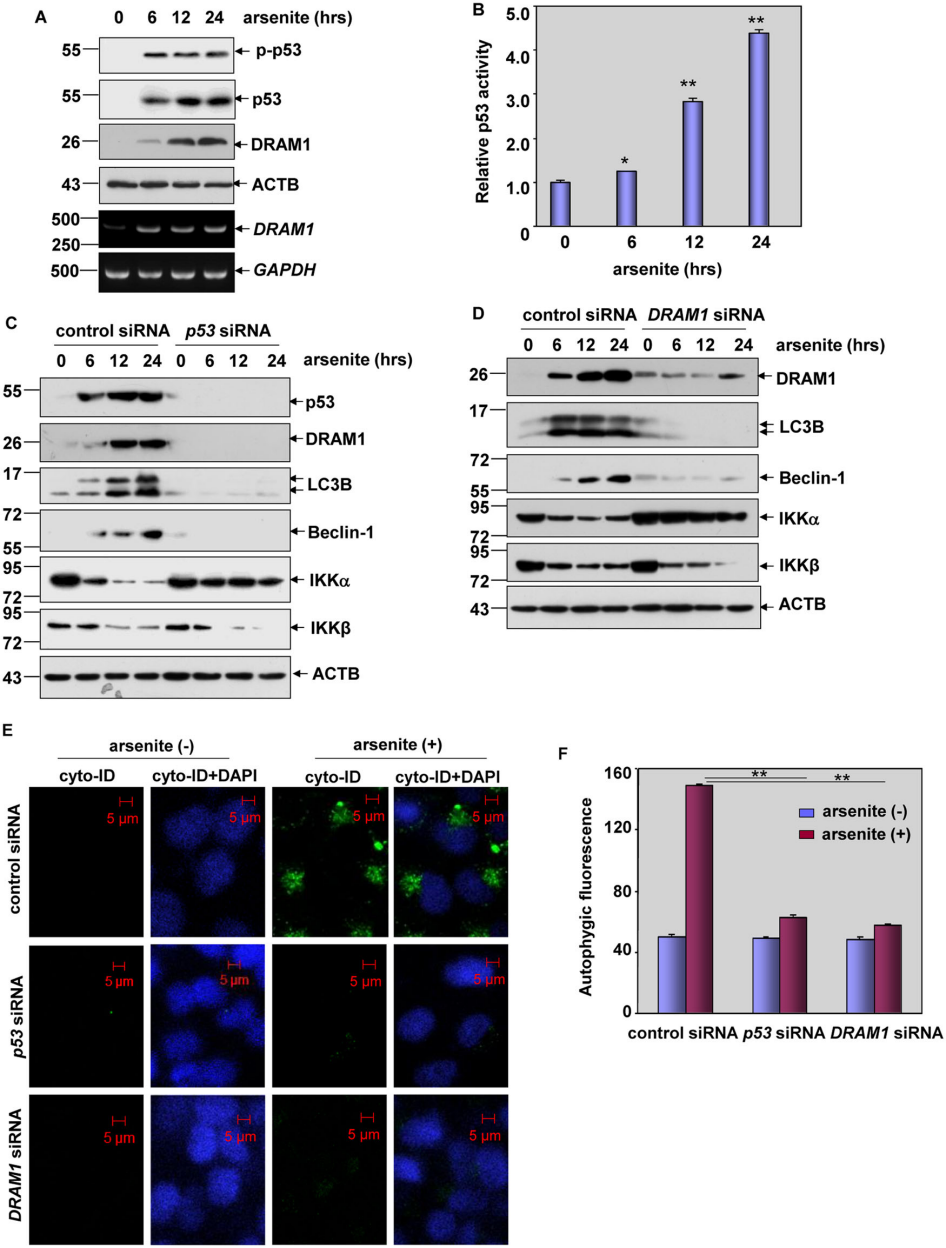
p53 pathway activation was responsible for autophagy-dependent degradation of IKKα under arsenite exposure. **a** HepG2 cells were treated with arsenite (20 μM) for the indicted time periods, and then the accumulation and activation of p53 and expression of DRAM1 were examined. **b** HepG2 cells were transfected with p53-dependent luciferase reporter, and the stable transfectants were established. The transfectants were exposed to arsenite (20 μM) for the indicted time periods, and the induction of p53-dependent luciferase activity was examined (*P < 0.05, **P < 0.01). **c**, **d** HepG2 cells were transfected with *p53* siRNA, *DRAM1* siRNA or control siRNA followed by exposure to arsenite (20 μM) 36 h after transfection. The activation status of p53/DRAM1 pathway, the induction of autophagy hallmarks and the expression levels of both IKKα and IKKβ were examined at 12 h after arsenite exposure. **e**, **f** HepG2 cells were transfected and treated as described in (**c** and **d**), and then the autophagy signals were detected as described in Fig. 2c, d (**P < 0.01).

In the following study, we focused on exploring the signaling events leading to selective degradation of IKKα after arsenite treatment. p53 is one of the important transcription factors that is involved in the modulation of autophagy^19^. In the arsenite-treated HepG2 cells, we observed a time-dependent accumulation of p53 and upregulation of p53 phosphorylation at serine 15 (Fig. 4a), a representative signaling event indicating the activation of this protein. Under the same conditions, an enhancement of p53-dependent luciferase activity was readily detected in HepG2 cells after arsenite exposure (Fig. 4b), further confirming the elevation of p53 transcriptional activity in response to arsenite. DRAM1 is the transcriptional target of p53 that functions as a positive regulator of autophagy^20^. As shown in Fig. 4a, we readily observed an obvious upregulation of DRAM1 transcription and protein synthesis accompanying by p53 transactivation after arsenite exposure. These data indicate that arsenite treatment effectively induces p53/DRAM1 pathway activation in HepG2 cells.

Next, *p53* and *DRAM1* siRNAs were separately transfected into HepG2 cells, then we observed that the upregulation of both LC3B and Beclin-1 upon arsenite exposure were almost completely blocked by knockdown of p53 or DRAM1 expression (Fig. 4c, d). Moreover, an obvious reduction in the autophagic fluorescence signals from the Cyto-ID-stained HepG2 cells with the impairment of p53 or DRAM1 expression was also detected (Fig. 4e, f), indicating that p53/DRAM1 pathway activation is essential for inducing autophagy in the arsenite-treated HepG2 cells. Most importantly, degradation of IKKα was totally blocked by interrupting p53 or DRAM1 expression, while transcriptional suppression of IKKβ did not affected under the same conditions (Fig. 4c, d). These data clearly demonstrate that p53 transactivation and DRAM1 induction are responsible for autophagy-dependent degradation of IKKα under arsenite exposure.

### The presence of IKKα was critical for p53 transactivation in response to arsenite exposure

**Fig. 5:**
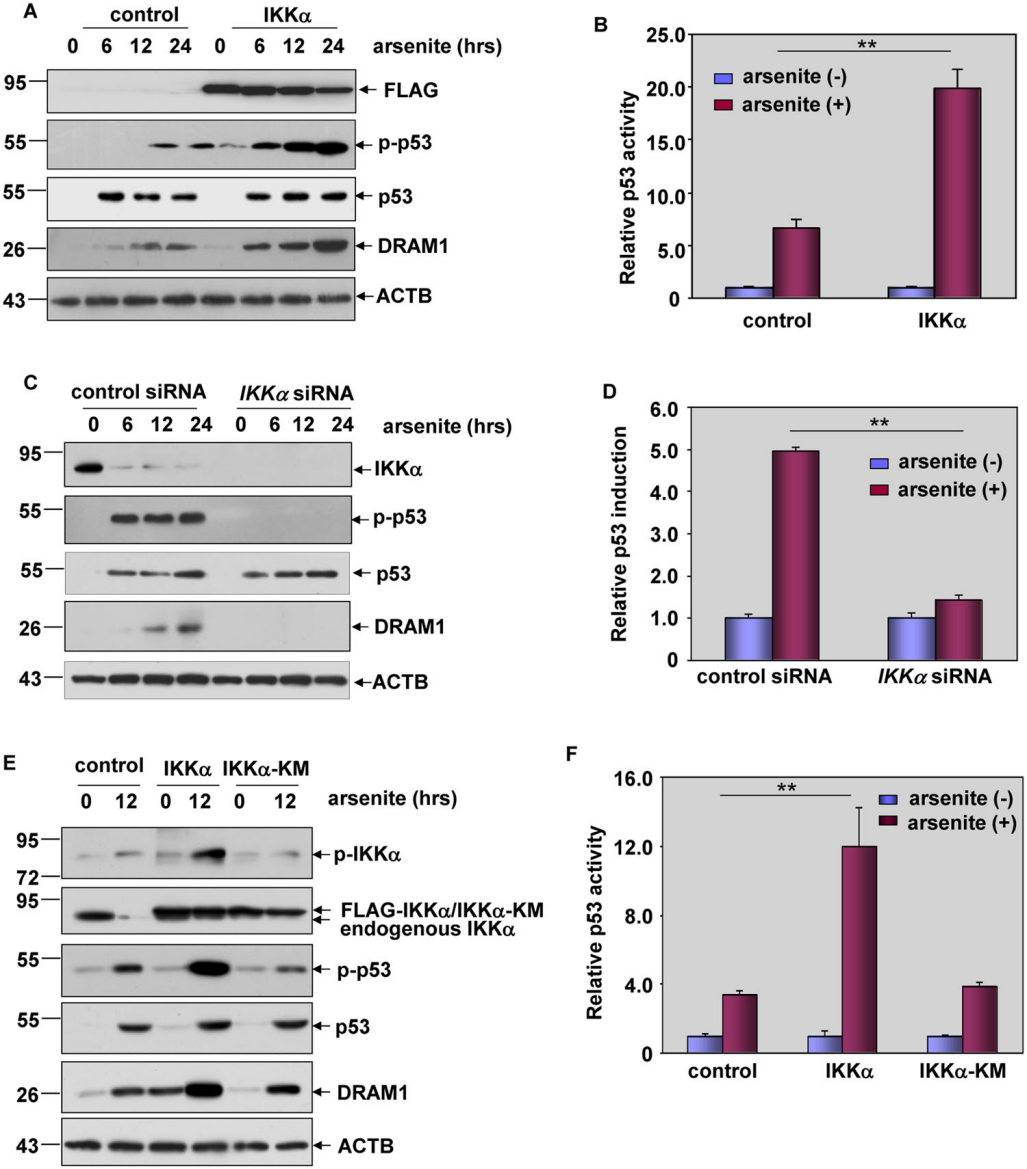
The presence of IKKα was critical for p53 transactivation in response to arsenite exposure. **a** HepG2 cells were transfected with expression plasmids encoding FLAG-IKKα or the control vector and then subjected to arsenite (20 μM) exposure. The activation of p53/DRAM1 pathway was detected at 12 h after arsenite treatment. **b** HepG2 cells stably transfected with p53-dependent luciferase reporter were transfected with FLAG-IKKα expression plasmid or the control vector and then exposed to arsenite (20 μM). The induction of p53-dependent luciferase activity was determined at 12 h after arsenite exposure. **c** HepG2 cells were transfected with siRNA specifically targeting *IKKα* or the control siRNA and then treated as described in (**a**). The detections were also performed as described in (**a**). **d** HepG2 cells stably transfected with p53-dependent luciferase reporter were transfected with *IKKα* siRNA or the control shRNA and then exposed to arsenite (20 μM). The induction of p53-dependent luciferase activity was determined as described in (**b**). **e** HepG2 cells were transfected with the expression plasmids encoding FLAG-IKKα, FLAG-IKKα-KM or the control vector. The phosphorylation of IKKα and activation of p53/DRAM1 pathway were detected at 12 h after arsenite exposure. **f** HepG2 cells stably transfected with p53-dependent luciferase reporter were transfected with expression plasmids encoding FLAG-IKKα, FLAG-IKKα-KM or the control vector and then exposed to arsenite (20 μM). The induction of p53-dependent luciferase activity was determined as described in (**b**).

Next, we focused on identifying the upstream protein kinase(s) responsible for p53-dependent autophagy induction in response to arsenite stimulation. As shown in Fig. 5a, a remarkably increase in p53 phosphorylation and DRAM1 expression was observed in the IKKα-overexpressed HepG2 cells after arsenite treatment. Moreover, we also found a significant upregulation of p53-dependent luciferase activity after overexpression of IKKα in HepG2 cells (Fig. 5b). On the contrary, knockdown of IKKα expression resulted in the complete inhibition of the p53 phosphorylation and DRAM1 upregulation induced by arsenite (Fig. 5c). In addition, an attenuation of p53-dependent luciferase activity was readily detected in HepG2 cells transfected with *IKKα* siRNA (Fig. 5d). Together, these data indicate that IKKα plays a critical role in mediating p53/DRAM1 pathway activation in the arsenite-induced response.

To address whether IKKα kinase activity is required for regulating p53/DRAM1 pathway activation in the arsenite response, wild-type IKKα or its kinase mutant, IKKα-KM, was transfected into HepG2 cells, respectively. We found that IKKα-KM lost the ability to be phosphorylated after arsenite exposure and failed to enhance p53 activation and DRAM1 induction as the wild-type IKKα did in HepG2 cells (Fig. 5e). Moreover, the effect of IKKα on promoting p53-dependent luciferase activity was totally abolished by abrogating its kinase activity (Fig. 5f). Therefore, we conclude that IKKα kinase activity is required for regulating p53/DRAM1 pathway activation in response to arsenite stimulation.

### The presence of IKKα was critical for autophagy induction in response to arsenite exposure

**Fig. 6:**
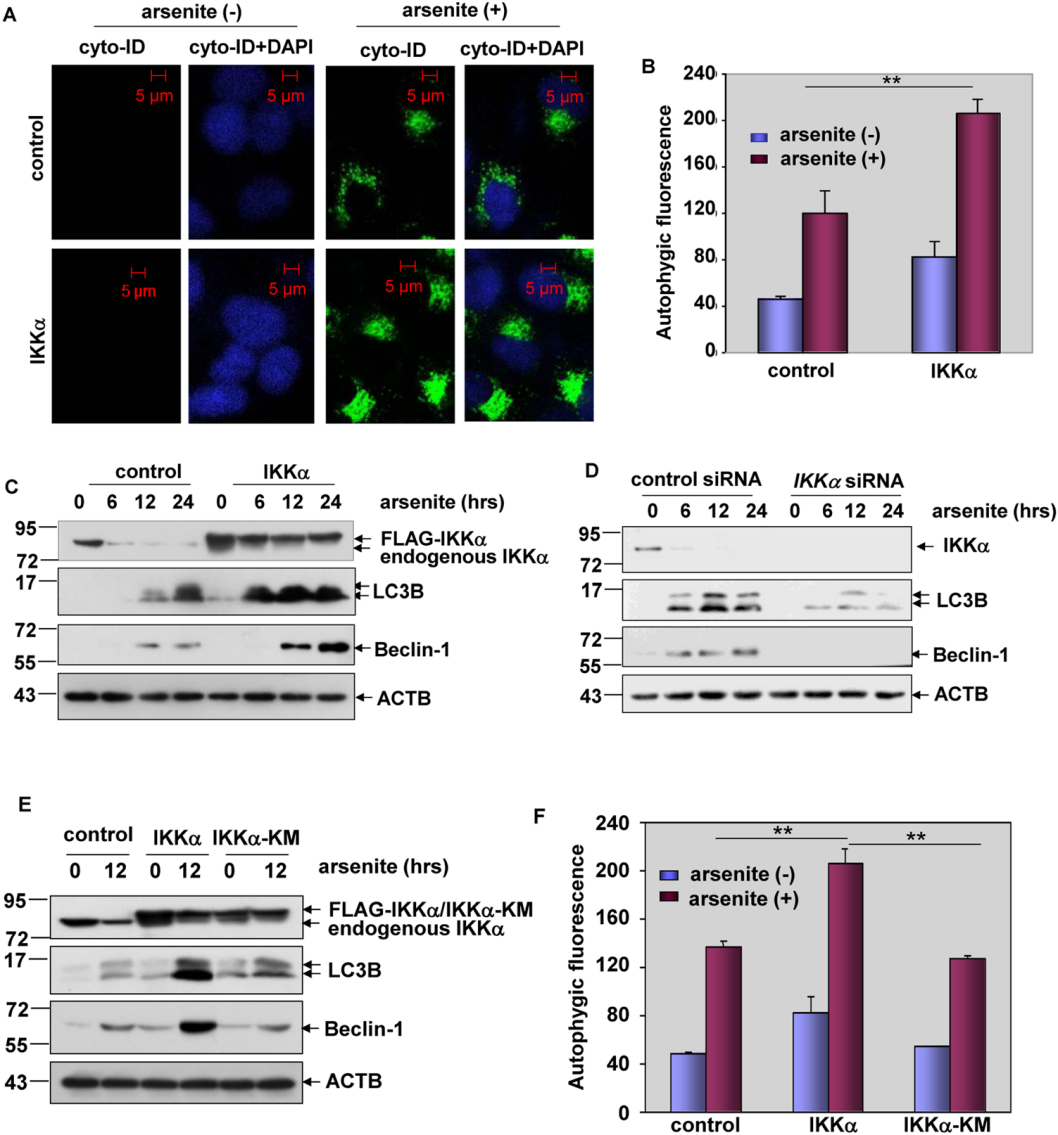
The presence of IKKα was critical for autophagy induction in response to arsenite exposure. **a**, **b** HepG2 cells were transfected with expression plasmids encoding FLAG-IKKα or the control vector and then subjected to arsenite (20 μM) exposure. The autophagy signals were detected as described in Fig. 2c, d (**P < 0.01). **c** HepG2 cells were transfected and treated with arsenite as described in (**a**, **b**), and then the expression levels of LC3B and Beclin-1 were detected at the indicated time periods after arsenite exposure. **d** HepG2 cells were transfected with siRNA specifically targeting *IKKα* or the control siRNA and then treated as described in (**a**). The detections were performed as described in (**c**). **e** HepG2 cells were transfected with the expression plasmids encoding FLAG-IKKα, FLAG-IKKα-KM or the control vector. The detections were performed as described in (**c**). **f** HepG2 cells were transfected and treated as described in (**e**). The autophagy signals were detected quantitatively as described in Fig. 2d (**P < 0.01).

We next investigated whether IKKα was also responsible for autophagy induction in response to arsenite stimulation. As shown in Fig. 6a, overexpression of IKKα in HepG2 cells significantly enhanced the synthesis of autophagosome, evidenced by the stronger green autophagic fluorescence signal accumulating in the perinuclear region of HepG2 cells. Furthermore, the flow cytometry-based quantitative analysis showed that the Cyto-ID-dependent autophagic fluorescence signals increased dramatically in IKKα-overexpressed HepG2 cells after exposure to arsenite (Fig. 6b). In addition, dramatic upregulation of arsenite-induced LC3B and Beclin-1 accumulation was also detected in HepG2 cells after overexpression of IKKα (Fig. 6c). On the contrary, induction of LC3B and Beclin-1 accumulation was almost totally blocked after knocking down IKKα expression (Fig. 6d). These data indicate that IKKα is also critical for autophagy induction in the arsenite responses. In the following study, we observed that abrogating the kinase activity of IKKα resulted in a complete loss of its function in enhancing LC3B and Beclin-1 accumulation (Fig. 6e). In addition, the increase in the Cyto-ID-dependent autophagic fluorescence signals by IKKα overexpression could not be observed in the IKKα-KM-overexpressed HepG2 cells under the same arsenite exposure conditions (Fig. 6f). Therefore, we conclude that IKKα kinase activity is also required for inducing p53-dependent autophagy in response to arsenite stimulation.

### IKKα activated p53/DRAM1/autophagy pathway through CHK1-dependent manner in response to arsenite exposure

**Fig. 7:**
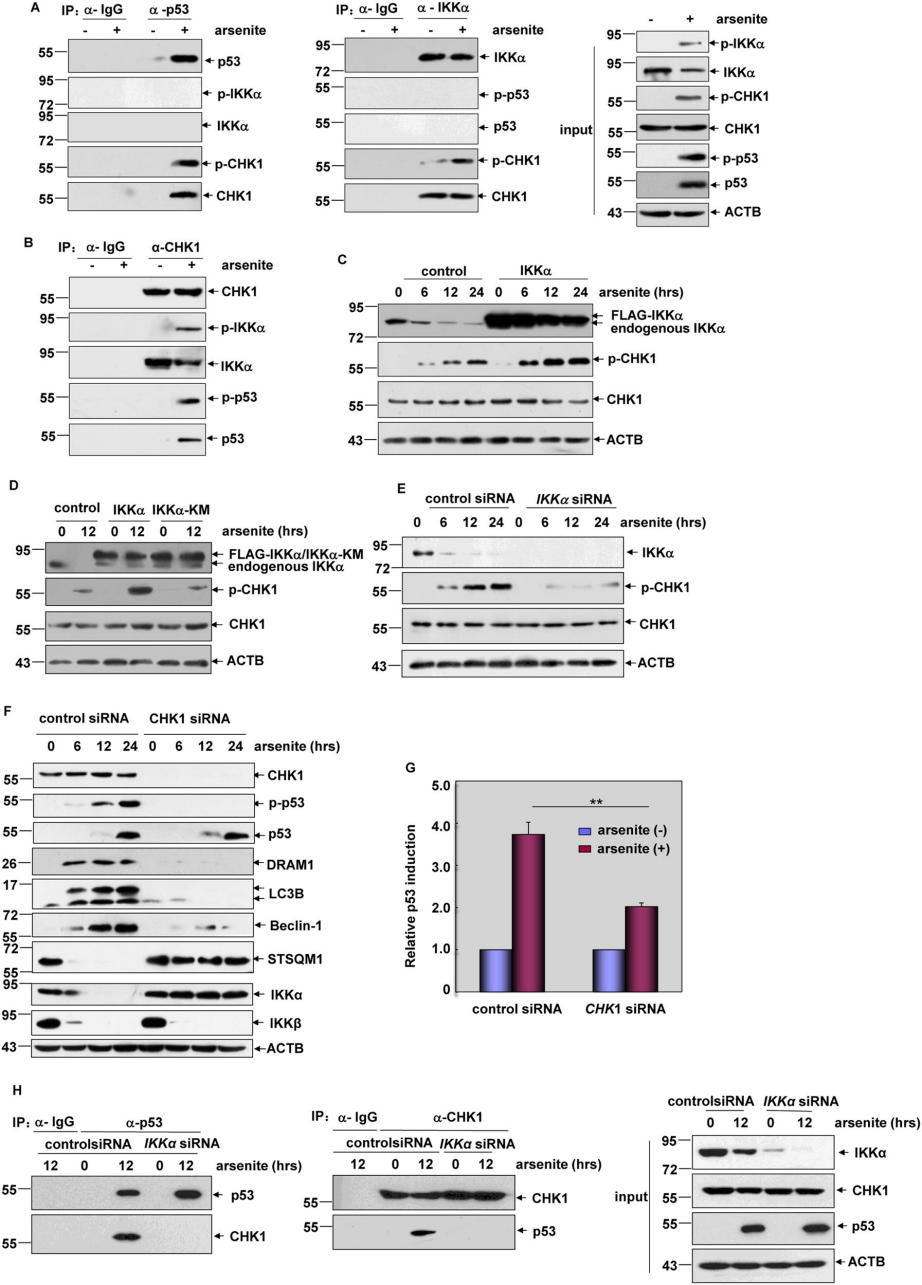
IKKα activated p53 through CHK1-dependent manner in response to arsenite exposure. **a** HepG2 cells were left untreated or treated with arsenite (20 μM). Cell lysate were immunoprecipitated with anti-p53 or anti-IKKα antibody or the control IgG, and then the immunoprecipitants were probed with the antibodies as indicated. **b** Cell lysate described in (**a**) were immunoprecipitated with anti-CHK1 antibody or the control IgG, and then the immunoprecipitants were probed with the antibodies as indicated. **c** HepG2 cells were transfected with expression plasmids encoding FLAG-IKKα or the control vector and then subjected to arsenite (20 μM) exposure. The activation status of CHK1 was detected at the indicated time periods after arsentie exposure. **d** HepG2 cells were transfected with the expression plasmids encoding FLAG-IKKα, FLAG-IKKα-KM or the control vector. The detection was performed as describe in (**c**). **e** HepG2 cells were transfected with siRNA specifically targeting *IKKα* or the control siRNA and then then subjected to arsenite (20 μM) exposure. The detection was performed as describe in (**c**). **f** HepG2 cells were transfected with siRNA specifically targeting *CHK1* or the control siRNA and then then subjected to arsenite (20 μM) exposure. The activation of p53/DRAM1/autophagy pathway and the expressions of IKKα and IKKβ were detected at the indicated time periods after arsentie exposure. **g** HepG2 cells stably transfected with p53-dependent luciferase reporter were transfected with *CHK1* siRNA or the control siRNA and then exposed to arsenite (20 μM). Then the induction of p53-dependent luciferase activity was determined (**P < 0.01). **h** HepG2 cells were transfected with *IKKα* siRNA or the control siRNA and then then subjected to arsenite (20 μM) exposure. Cell lysate were immunoprecipitated with anti-p53 or anti-CHK1 antibody or the control IgG, and then the immunoprecipitants were probed with the antibodies as indicated.

Although IKKα was phosphorylated upon arsenite exposure and its kinase activity was required for p53/DRAM1/autophagy pathway activation, we did not find the interaction between IKKα and p53 in the arsenite responses (Fig. 7a). This result suggests that IKKα might be involved in p53 transactivation and the subsequent autophagy induction through an indirect manner. Among the previous identified protein kinases that contribute to p53 activation^21^, CHK1 was observed to constitutively interact with IKKα in resting HepG2 cells and the binding ability of the activated form of CHK1 and IKKα was significantly enhanced after arsenite stimulation (Fig. 7a, b). Furthermore, induced interaction between the CHK1 and p53 was also detected in the arsenite-treated HepG2 cells (Fig. 7a, b). These data indicate that CHK1 might be involved in mediating IKKα-dependent p53 activation. Then we found that CHK1 activation induced by arsenite was significantly upregulated by IKKα but not IKKα-KM overexpression (Fig. 7c, d). On the contrary, knocking down IKKα levels resulted in dramatic suppression on CHK1 activation (Fig. 7e). These results indicate that CHK1 functions as a downstream target of IKKα in the arsenite responses. In the following study, we further observed the suppression of p53 phosphorylation and activation, DRAM1 upregulation and autophagy induction in HepG2 cells with the impairment of CHK1 expression (Fig. 7f). Moreover, arsenite-induced p53-depednent luciferase activity was dramatically inhibited by knocking down CHK1 expression (Fig. 7g). Most importantly, degradation of IKKα was totally blocked in HepG2 cells in the absence of CHK1 expression, while suppression on IKKβ expression did not change under the same conditions (Fig. 7f). These data clearly demonstrate the critical role of CHK1 in mediating p53/DRAM1/autophagy-dependent IKKα degradation in the arsenite responses. Finally, we found that knockdown IKKα expression totally blocked the induced interaction of CHK1 with p53 (Fig. 7h), indicating that the presence of IKKα is required for CHK1/p53 complex formation in HepG2 cells treated with arsenite.

Taken these data together, we conclude that arsenite exposure induces the activation of IKKα, which activates p53/DRAM1/autophagy pathway by promoting CHK1 phosphorylation and CHK1/p53 interaction. Then, the activated IKKα can be specifically recognized by the autophagy machinery via directly binding with LC3B, leading to degradation of IKKα and cell apoptosis.

## Discussion

With the discovery of more and more NF-κB-unrelated signaling molecules whose activities are regulated by IKKα or IKKβ, the biological roles for these two catalytic subunits of IKK complex have gone far beyond NF-κB activation^10–12^. Although the reports are limited, regulating autophagy has been revealed to be a NF-κB-independent function of IKKs^22–25^. For example, in response to nutrient starvation, both IKKα and IKKβ are involved in inducing autophagy by upregulating essential autophagic genes expression (LC3B, Beclin-1 and ATG5)^22^, alleviating activities of the autophagy inhibitors (p53, mTOR)^23,24^, or connecting to the canonical autophagy induction pathways (AMPK and JNK1) activation^23^. While in mammary epithelial cells (MECs) deprived of extracellular matrix contact, IKKβ is proved to be required for stimulating cytoprotective autophagy to antagonize anoikis via NF-κB-independent manner, although the precise mechanism remains elusive^25^. In this study, we have revealed the new functional link between IKKα and autophagy induction in response to the treatment of cytotoxic chemical reagent, arsenite. Moreover, this effect of IKKα was unrelated to NF-κB activity, but was delivered by inducing p53 pathway activation. Interestingly, although IKKα and its kinase activity were required for p53 transactivation, no direct interaction between IKKα and p53 was observed under arsenite exposure. In the attempt to identify the adaptor linking IKKα and p53 activation, we found that CHK1 functioned as the downstream target of IKKα to trigger p53-dependent autophagy after arsenite exposure. These results thus have provided novel mechanistic findings regarding NF-κB-unrelated function of IKK in regulating autophagy by cross talking with p53.

In addition to eliciting autophagy, IKKα could also be selectively recognized by the autophagic machinery after activation, and this feed-back selective degradation of IKKα is critical for mediating the pro-apoptotic effect of arsenite. As far as we know, this is the first report regarding the mechanism of IKKα degradation by selective autophagy under stress condition. Usually, intracellular proteins undergo selective autophagic degradation via directly binding with LC3B or other ATG8 family members or by linking to the cargo receptors^6–9^. IKKβ degradation by selective autophagy under both steady state and pro-inflammatory conditions have been reported in the previous studies, which requires monoubiquitination of IKKβ^26,27^ or binding with cargo adaptor or receptor to deliver IKKβ into the autophagosome^28–30^. However, we neither observed the ubiquitination of IKKα and IKKβ nor any dynamic changes on IKKα and IKKβ expression with or without the expressions of the well-known cargo receptors (p62, NBR1, etc.) (data not shown). Under the same conditions, direct IKKα-LC3B interaction was observed. However, although two sequences perfectly matched to property of the classical LIR motif (W/F/Y-X-X-V/L/I) were identified within the kinase domain of IKKα, neither of the classical LIR motifs within IKKα possessed the function in mediating LC3B interaction, while Ala 216 within the N-terminal arm of the first putative LIR played a critical role in this process. This result also perfectly explains why IKKβ, which possesses the exactly same two LIR motifs as IKKα, is unable to interact with LC3B and fails to be identified by the autophagic machinery in the arsenite-induced responses.

Multiple data support that in addition to the aromatic residue (W/F/Y) in the first position and the large, hydrophobic residue (V/L/I) in the fourth position of LIR core sequence, the importance of an acidic charge (E, D, S or T), either N- or C-terminal to the conserved aromatic residue in LIR of the cargo, in mediating selective autophagy is evident^6,31^. And the function of these acid residues is to provide negative charge in mediating the electrostatic interaction with the positive charge of the basic residues in LC3 (R10, R11, K30, K49 and K50)^6,31,32^. However, according to our results, the structural basis determining IKKα-LC3B interaction was quite different. Since mutation on a single nonpolar amino acid (Ala 216) in IKKα totally abolished its LC3B binding ability, we speculated that electrostatic interaction might not be essential to mediate the binding of these two molecules. To further address this issue, we are currently working on determining whether the binding affinity of IKKα to wild-type LC3B and its mutants with the basic residues mutation (R10, R11, K30, K49, and K50) is different. Additionally, we noticed that the amino acid in IKKβ corresponding to Ala 216 in IKKα is Thr 217. Therefore, whether IKKβ undergoes phosphorylation on Thr 217 and then results to the prevention of IKKβ-LC3B interaction due to the space hindrance in the arsenite-induced responses needs to be further clarified.

In our recent published report, we have revealed p53-dependent transcriptional repression on *IKKβ* under arsenite exposure^17^. In fact, the putative p53-responsive elements (p53-REs) were identified within both the *IKKα* and *IKKβ* promoter regions. However, only recruitment of the activated p53 to p53-RE within the *IKKβ* promoter was observed in HepG2 cells after arsenite exposure; no *IKKα* promoter chromatin-associated p53 was detected under the same conditions^17^. Therefore, not only the autophagy-dependent degradation but also the transcriptional regulation showed selectivity toward IKKα and IKKβ. In the previous reports, including our own, multiple evidence support that the specificity of the two catalytic subunits of IKK in regulating signaling events unrelated to NF-κB exists under a variety of stress conditions^10–16^. Data in the current study and our recently published work^17^ further disclosed the different upstream mechanism involving in the specific regulation on IKKα or IKKβ expression. Taken these data together, we believe that both IKKα and IKKβ have the ability of cross-talking with multiple signaling events far beyond NF-κB activation to determine cell fate.
